# Dyslexia and language impairment associated genetic markers influence cortical thickness and white matter in typically developing children

**DOI:** 10.1007/s11682-015-9392-6

**Published:** 2015-05-09

**Authors:** John D. Eicher, Angela M. Montgomery, Natacha Akshoomoff, David G. Amaral, Cinnamon S. Bloss, Ondrej Libiger, Nicholas J. Schork, Burcu F. Darst, B. J. Casey, Linda Chang, Thomas Ernst, Jean Frazier, Walter E. Kaufmann, Brian Keating, Tal Kenet, David Kennedy, Stewart Mostofsky, Sarah S. Murray, Elizabeth R. Sowell, Hauke Bartsch, Joshua M. Kuperman, Timothy T. Brown, Donald J. Hagler, Anders M. Dale, Terry L. Jernigan, Jeffrey R. Gruen, on behalf of the Pediatric Imaging Neurocognition Genetics study

**Affiliations:** Department of Genetics, Yale University, New Haven, CT 06520 USA; Department of Pediatrics, Yale University School of Medicine, New Haven, CT 06520 USA; Center for Human Development, University of California, La Jolla, San Diego, CA 92037 USA; Department of Psychiatry, University of California, La Jolla, San Diego, CA 92037 USA; Department of Psychiatry and Behavioral Sciences, University of California, Davis, CA 95817 USA; Scripps Genomic Medicine, Scripps Health, Scripps Translational Science Institute, La Jolla, CA 92037 USA; Sackler Institute for Developmental Psychobiology, Weil Cornell Medical College, New York, NY 10065 USA; Department of Medicine, Queen’s Medical Center, University of Hawaii, Honolulu, HI 96813 USA; Department of Psychiatry, University of Massachusetts Medical School, Boston, MA 01655 USA; Kennedy Krieger Institute, 707 N. Broadway, Baltimore, MD 21205, USA; Department of Neurology, Harvard Medical School, Children’s Hospital Boston, Boston, MA 02115 USA; Department of Neurology and Athinoula A. Martinos Center for Biomedical Imaging, Massachusetts General Hospital, Charlestown, MA 02129 USA; Department of Pediatrics, University of Southern California, Los Angeles, CA 90027 USA; Developmental Cognitive Neuroimaging Laboratory Children’s Hospital, Los Angeles, CA 90027 USA; Multimodal Imaging Laboratory, University of California, La Jolla, San Diego, CA 92037 USA; Department of Neurosciences, University of California, La Jolla, San Diego, CA 92037 USA; Radiology University of California, La Jolla, San Diego, CA 92037 USA; Cognitive Science University of California, La Jolla, San Diego, CA 92037 USA; Department of Investigative, School of Medicine, Medicine Yale University, New Haven, CT 06520 USA; Department of Pediatrics, Genetics, and Investigative Medicine, Yale Child Health Research Center, 464 Congress Avenue, New Haven, CT 06520-8081 USA

**Keywords:** Dyslexia, Language impairment, *KIAA0319*, DYX3, DYX2, Imaging-genetics

## Abstract

Dyslexia and language impairment (LI) are complex traits with substantial genetic components. We recently completed an association scan of the DYX2 locus, where we observed associations of markers in *DCDC2*, *KIAA0319*, *ACOT13*, and *FAM65B* with reading-, language-, and IQ-related traits. Additionally, the effects of reading-associated DYX3 markers were recently characterized using structural neuroimaging techniques. Here, we assessed the neuroimaging implications of associated DYX2 and DYX3 markers, using cortical volume, cortical thickness, and fractional anisotropy. To accomplish this, we examined eight DYX2 and three DYX3 markers in 332 subjects in the Pediatrics Imaging Neurocognition Genetics study. Imaging-genetic associations were examined by multiple linear regression, testing for influence of genotype on neuroimaging. Markers in DYX2 genes *KIAA0319* and *FAM65B* were associated with cortical thickness in the left orbitofrontal region and global fractional anisotropy, respectively. *KIAA0319* and *ACOT13* were suggestively associated with overall fractional anisotropy and left pars opercularis cortical thickness, respectively. DYX3 markers showed suggestive associations with cortical thickness and volume measures in temporal regions. Notably, we did not replicate association of DYX3 markers with hippocampal measures. In summary, we performed a neuroimaging follow-up of reading-, language-, and IQ-associated DYX2 and DYX3 markers. DYX2 associations with cortical thickness may reflect variations in their role in neuronal migration. Furthermore, our findings complement gene expression and imaging studies implicating DYX3 markers in temporal regions. These studies offer insight into where and how DYX2 and DYX3 risk variants may influence neuroimaging traits. Future studies should further connect the pathways to risk variants associated with neuroimaging/neurocognitive outcomes.

**Electronic supplementary material:**

The online version of this article (doi:10.1007/s11682-015-9392-6) contains supplementary material, which is available to authorized users.

## Introduction

Neurocognitive and language traits are complex phenotypes with substantial environmental and genetic components. Specifically, dyslexia (also known as reading disability or RD) and language impairment (LI) as well as quantitative performance in reading, language, and cognitive skills are heritable traits, with heritability estimates ranging from 45 to 84 % (DeFries et al. 1987; Bishop and Hayiou-Thomas 2008; Pennington and Bishop 2009; van Soelen et al. 2011). RD is characterized by unexpected difficulties in reading despite normal child development as well as adequate educational instruction and opportunity (Pennington and Bishop 2009). Children with LI generally have unexplained difficulties in oral language, as opposed to written language deficits seen in RD, despite normal child development and adequate opportunity (Pennington and Bishop 2009; Newbury et al. 2010). Both RD and LI adversely affect a child’s academic, linguistic, and social development and can hamper academic achievement.

Several candidate loci and genes for RD and LI have been identified. Two of these candidate regions are the DYX2 locus on chromosome 6p22 and the DYX3 locus on chromosome 2p12. Both DYX2 and DYX3 were identified through linkage studies of families with RD (Fagerheim et al. 1999; Anthoni et al. 2007; Kaminen et al. 2003; Cardon et al. 1994; Gayán et al. 1999; Kaplan et al. 2002; Deffenbacher et al. 2004). The DYX2 locus is the most replicated RD risk locus, with subsequent association studies identifying two well-established risk genes, *DCDC2* and *KIAA0319* (Meng et al. 2005; Schumacher et al. 2006; Harold et al. 2006). Following their associations with RD, other studies have shown that genes within the DYX2 locus contribute to quantitative reading and language performance as well as other related neurocognitive and language traits including LI, overall cognition, and speech sound disorder (SSD) (Scerri et al. 2011; Powers et al. 2013; Eicher et al. 2014; Smith et al. 2005; Newbury et al. 2011). Our group recently reported the results of an association scan across the entire DYX2 locus with reading, language, and cognitive traits in an unselected, population-based sample from the United Kingdom (Powers et al. 2013; Eicher et al. 2014). There, we recapitulated the associations of *DCDC2* and *KIAA0319* as well as implicated two new candidate genes *FAM65B* and *CMAHP*. Markers within *ACOT13* (also known as *THEM2*) and *C6orf62* also were associated with these traits but were in linkage disequilibrium (LD) with a previously identified risk haplotype in *KIAA0319*, leading us to hypothesize that the associations of *ACOT13* and *C6orf62* tagged variation in *KIAA0319* (Eicher et al. 2014; Francks et al. 2004; Paracchini et al. 2006).

The DYX3 locus on chromosome 2p12 is less studied than the DYX2 locus. Two candidate genes, *MRPL19* and *GCFC2* (also referred to as *C2orf3*), have been proposed with mixed results in replication analyses for each (Fagerheim et al. 1999; Anthoni et al. 2007; Kaminen et al. 2003; Peyrard-Janvid et al. 2004; Paracchini et al. 2011; Scerri et al. 2012). Similar to DYX2, the DYX3 locus appears to contribute not only to RD and reading-related traits but other neurocognitive domains and disorders. For instance, Scerri et al. found association of markers within the *MRPL19/GCFC2* locus with both verbal and performance IQ (Scerri et al. 2012) in a population-based European sample. Furthermore, a genome-wide association study (GWAS) showed a suggestive association of a marker in *GCFC2* with Alzheimer’s disease (Melville et al. 2012). Although not exactly related to pediatric language disorders, this suggestive association with Alzheimer’s disease, a late-onset disorder with neurocognitive impairments, further implicates a possible general role of *GCFC2* in overall neurocognitive skills as well as language-related domains.

These studies have largely focused on the genetic relationship with neurobehavioral measures of reading, language, and cognition, as these are the instruments used clinically. However, solely using neurobehavioral measures does not necessarily give insight into the underlying molecular and neurological mechanisms of these traits. Intermediate phenotypes, that represent biological phenomena closer to the genetic function, can provide a powerful approach to gain insight into pathophysiology (Thompson et al. 2010). Therefore, to gain further insight into neurological mechanism, in vivo neuroimaging techniques can reveal structural, connectivity, and functional implications of genes and associated genetic variants (Eicher and Gruen 2013; Graham and Fisher 2013).

In this vein, human imaging-genetics studies have examined the relationships of DYX2 and DYX3 risk variants with various neuroimaging modalities. *DCDC2* variants have been associated with overall grey matter volume as well as in superior prefrontal, temporal, and occipital networks (Meda et al. 2008; Jamadar et al. 2011). *KIAA0319* markers were also associated with gray matter in the superior and inferior cerebellar networks (Jamadar et al. 2011). Darki et al. reported the association of *DCDC2* and *KIAA0319* markers with left temporo-parietal white matter volume (Darki et al. 2012). Left temporo-parietal white matter volumes were then associated with reading skills in the same subjects, suggesting possible mediation between risk genetic markers and behavioral outcome (Darki et al. 2012). In addition to volumetric imaging measures, *DCDC2* has also been associated with brain activation patterns during reading-related tasks using functional magnetic resonance imaging (fMRI) (Cope et al. 2012). *ACOT13*, which may tag variation in *KIAA0319*, was associated with asymmetry in activation of the superior temporal sulcus during reading tasks (Pinel et al. 2012). The observed lower asymmetry of brain activation patterns was similar to the increased bilateral symmetry in brain activation seen in impaired school-age child readers (Brown et al. 2005). These initial studies have started to provide insight into the neuroimaging implications of behaviorally associated *DCDC2* and *KIAA0319* markers. However, further study and replication analyses are needed to understand the connection between risk genes, neuroimaging implications, and the ultimate neurobehavioral phenotype.

DYX3 candidate risk genes *MRPL19* and *GCFC2* are highly co-expressed in brain regions implicated in reading processes, including the inferior frontal and temporal occipital regions as well as the superior temporal, parietal temporal and middle temporal gyri (Anthoni et al. 2007). Furthermore, expression of *MRPL19* and *GCFC2* are correlated strongly with other RD candidate genes, including *DYX1C1, DCDC2, KIAA0319* and *ROBO1* (Anthoni et al. 2007). Associated markers in the *MRPL19/GCFC2* locus were related with white matter in the posterior corpus callosum and cingulum, regions that connect large portions of the parietal, occipital and temporal lobes (Scerri et al. 2012). Furthermore, a recent GWAS showed an association of *GCFC2* with hippocampal volume (Melville et al. 2012). These initial studies point to *MRPL19* and *GCFC2* influencing cortical measures and white matter volumes in temporal and hippocampal regions, which then may influence language and neurocognitive traits. However, more work is needed to replicate and substantiate these findings in independent studies.

The overall goal of this study is to examine the neuroimaging implications of DYX2 and DYX3 markers previously associated with reading, language, and/or IQ. To accomplish this, we utilize genetic and neuroimaging data collected in typically developing children in the Pediatric Imaging Neurocognition Genetics (PING) study. First, using the information gained by our recent association scan of the DYX2 locus in an unselected sample, we examine the replicated markers with cortical thickness and volume using structural magnetic resonance imaging (sMRI) and fractional anisotropy (FA) using diffusion tensor imaging (DTI) (Powers et al. 2013; Eicher et al. 2014). Second, we perform association analyses of DYX3 markers previously associated with neuroimaging measures in hippocampal and temporal regions (Scerri et al. 2012; Melville et al. 2012). Here, we aim to confirm the contribution of these markers to neuroimaging phenotypes in these cortical regions.

## Methods

### The pediatrics imaging neurocognition genetics (PING) study

Recruitment and experimental methods for the PING study are described in detail elsewhere, but are summarized briefly below (Akshoomoff et al. 2014; Brown et al. 2012; Fjell et al. 2012; Walhovd et al. 2012; Eicher et al. 2013). The PING study is a cross-sectional cohort of typically developing children between the ages of 3 and 20 years. Subjects were excluded for history of major developmental, psychiatric, and/or neurological disorders, brain injury, or medical conditions that affect neurological development. However, subjects were not excluded due to learning or language disabilities such as RD and LI. The human research protections programs and institutional review boards at the 10 institutions (Weil Cornell Medical College, University of California at Davis, University of Hawaii, Kennedy Krieger Institute, Massachusetts General Hospital, University of California at Los Angeles, University of California at San Diego, University of Massachusetts Medical School, University of Southern California, and Yale University) participating in the PING study approved all experimental and consenting procedures. For individuals under 18 years of age, parental informed consent and child assent (for those 7 to 17 years of age) were obtained. All participants age 18 years and older gave their written informed consent.

### PING imaging analyses

PING imaging techniques, data acquisition, and analyses are discussed in depth elsewhere and briefly below (Brown et al. 2012; Fjell et al. 2012; Walhovd et al. 2012). Across the ten sites and 12 scanners, a standardized multiple modality high-resolution sMRI protocol was implemented, involving 3D T1- and T2-weighted volumes and a set of diffusion-weighted scans. At the University of California at San Diego (UCSD), data were obtained on a GE 3T SignaHD× scanner and a 3T Discovery 750× scanner (GE Healthcare) using eight-channel phased array head coils. The protocol included a conventional three-plane localizer, a sagittal 3D inversion recovery spoiled gradient echo T1-weighted volume optimized for maximum gray/white matter contrast (echo time = 3.5 ms, repetition time = 8.1 ms, inversion time = 640 ms, flip angle = 8°, receiver bandwidth = ±31.25 kHz, FOV = 24 cm, frequency = 256, phase = 192, slice thickness = 1.2 mm), and two axial 2D DTI pepolar scans (30-directions bvalue = 1000, TE = 83 ms, TR = 13,600 ms, frequency = 96, phase = 96, slice thickness = 2.5 mm). Acquisition protocols with pulse sequence parameters identical or near identical to those protocols used at UCSD were installed on scanners at the other sites. Data were acquired on all scanners to estimate relaxation rates and measure and correct for scanner-specific gradient coil nonlinear warping. Image files in DICOM format were processed with an automated processing stream written in MATLAB (Natick, MA) and C++ by the UCSD Multimodal Imaging Laboratory. T1-weighted structural images were corrected for distortions caused by gradient non-linearities, co-registered, averaged, and rigidly re-sampled into alignment with an atlas brain. Image post-processing and analysis were performed using a fully automated set of tools available in the FreeSurfer software suite (http://surfer.nmr.mgh.harvard.edu/) as well as an atlas-based method for delineating and labeling white matter fiber tracts (Fischl 2012).

### DTI methods

Diffusion-weighted images were corrected for eddy current distortion using a least squares inverse and iterative conjugate gradient descent method to solve for the 12 scaling and translation parameters describing eddy current distortions across the entire diffusion MRI scan, explicitly taking into account the orientations and amplitudes of the diffusion gradient (Zhuang et al. 2006). Head motion was corrected by registering each diffusion-weighted image to a corresponding image synthesized from a tensor fit to the data (Hagler et al. 2009). Diffusion MRI data were corrected for spatial and intensity distortions caused by B0 magnetic field in-homogeneities using the reversing gradient method (Holland et al. 2010). Distortions caused by gradient nonlinearities were corrected by applying a predefined, scanner-specific, nonlinear transformation (Jovicich et al. 2006). Diffusion-weighted images were automatically registered to T1-weighted structural images using mutual information and rigidly re-sampled into a standard orientation relative to the T1-weighted images with isotropic 2-mm voxels (Wells et al. 1996). Cubic interpolation was used for all re-sampling steps. Conventional DTI methods were used to calculate diffusion measures (Basser et al. 1994; Pierpaoli et al. 1996). Scanning duration for the DTI sequence was 4:24 min. White matter fiber tracts were labeled using a probabilistic-atlas based segmentation method (Hagler et al. 2009). Voxels containing primarily gray matter or cerebral spinal fluid, identified using FreeSurfer’s automated brain segmentation were excluded from analysis (Fischl et al. 2002). Fiber tract volumes were calculated as the number of voxels with probability greater than 0.08, the value that provided optimal correspondence in volume between atlas-derived regions of interest (ROIs) and manually traced fiber tracts. Next, fractional anisotropy (FA) was calculated within these atlas-derived fiber ROIs for every subject.

### Genetics methods in PING

Subjects were genotyped on the Illumina Human660W-Quad BeadChip (San Diego, CA), with markers passing quality control filters (sample call rate > 98 %, SNP call rate > 95 %, minor allele frequency > 5 %). A reference panel for genetic ancestry was constructed as previously described (Brown et al. 2012; Fjell et al. 2012; Walhovd et al. 2012). To assess ancestry and admixture proportions, we used a supervised clustering approach implemented in the ADMIXTURE software and grouped participant data into six clusters corresponding to six major continental populations: African, Central Asian, East Asian, European, Native American, and Oceanic (Alexander et al. 2009; Brown et al. 2012; Fjell et al. 2012; Walhovd et al. 2012). To prevent possible population stratification and as past genetic associations with selected markers were in European populations, only subjects with a European genetic ancestry factor (GAF) of 1 were included in analyses.

Fourteen markers previously showed evidence of replicated association with reading-, language-, and/or IQ-related traits within the DYX2 locus (Powers et al. 2013; Eicher et al. 2014); of these, 7 were directly genotyped in the PING study (Table 1). Additionally, rs9461045, a putative functional SNP associated with expression of *KIAA0319*, was directly genotyped in PING, totaling 8 DYX2 markers for analysis (Dennis et al. 2009). Three DYX3 markers had previously been associated with neuroimaging phenotypes and were directly genotyped in the PING study (Table 1) (Scerri et al. 2012; Melville et al. 2012). Markers were coded as either (1) carriers versus non-carriers of the minor allele (minor allele frequency < 0.25) or (2) homozygous major allele versus heterozygous versus homozygous minor (minor allele frequency > 0.25), termed “Additive” (Table 1).

**Table 1:** DYX3 (Chromosome 2p12, *n* = 3) and DYX2 (Chromosome 6p22, *n* = 8) markers directly genotyped in the PING study that showed replicated association with RD, LI, and/or IQ/

SNP	Ch	BP	Min All	MAF	Gene	Associated traits	Coded As:
rs917235	2	75825819	G	0.339	N/A^a^	RD, IQ	Additive
rs6732511	2	75839733	A	0.095	N/A^a^	RD, IQ	Carrier vs. Non-carrier
rs2298948	2	75926565	G	0.172	*GCFC2*	Hippocampus	Carrier vs. Non-carrier
rs707864	6	24305848	G	0.117	*DCDC2*	RD, LI	Carrier vs. Non-carrier
rs9295626	6	24587339	A	0.214	*KIAA0319*	RD, LI, IQ	Carrier vs. Non-carrier
rs10456309	6	24589562	A	0.032	*KIAA0319*	RD, LI, IQ	Carrier vs. Non-carrier
rs4576240	6	24596478	A	0.117	*KIAA0319*	RD, LI, IQ	Carrier vs. Non-carrier
rs9461045	6	24649061	A	0.172	*KIAA0319*	RD, LI, IQ	Carrier vs. Non-carrier
rs3777663	6	24700235	G	0.215	*ACOT13*	LI, IQ	Carrier vs. Non-carrier
rs3756814	6	24705835	C	0.367	*C6orf62*	LI, IQ	Additive
rs9348646	6	24820219	G	0.306	*FAM65B*	IQ	Additive

*Ch* Chromosome, *Min All* Minor Allele, *MAF* Minor Allele Frequency, *RD* Reading Disability, *LI* Language impairment

^a^rs917235 and rs6732511 are located in the DYX3 locus upstream of *GCFC2* and *MRPL19*

### Imaging-genetics analysis

Imaging-genetics analyses were performed in individuals of European genetic ancestry (*n* = 332) with imaging measures and DYX2/DYX3 genotypes that passed quality control. Scanner, age, handedness, socioeconomic status, and sex were included as covariates in all analyses (Akshoomoff et al. 2014; Brown et al. 2012; Fjell et al. 2012; Walhovd et al. 2012; Eicher et al. 2013). Different ROIs and imaging modalities were chosen for DYX2 and DYX3 markers (Supplemental Tables 1–2). DYX2 markers were conditioned on FA and cortical thickness in 16 fiber tracts of interest and 15 ROIs, respectively (Supplemental Table 1). Fiber tracts of interest and ROIs were chosen for DYX2 associations based on their previous implications in language and reading. DYX2 markers were examined for fractional anisotropy (FA) in the following fiber tracts: All Fiber Tracts (All), Inferior Longitudinal Fasciculus (ILF), Inferior Fronto-occipital Fasciculus (IFO), Superior Longitudinal Fasciculus (SLF), Temporal Superior Longitudinal Fasciculus (tSLF), Parietal Superior Longitudinal Fasciculus (pSLF), and Striatal Inferior Frontal Cortex (SIFC) in both right and left hemispheres, as well as All Fiber Tracts and Corpus Callosum (CC) bilaterally (Supplemental Table 1). DYX2 cortical ROIs were selected using the genetically relevant cortical parcellations as described in Chen et al. (Chen et al. 2011, 2012). We chose to use these parcellations because they are more likely to show associations with genetic factors based on their previously explored genetic relationships (Chen et al. 2011, 2012). DYX2 markers were examined for cortical thickness in the following regions: Superior Parietal, Orbitofrontal, Superior Temporal, Inferior Parietal, Dorsomedial Frontal, Precuneus, Dorsolateral Prefrontal, Pars Opercularis, and Central in the left hemisphere, as well as Occipital, Anteromedial Temporal, and Posterolateral Temporal in both right and left hemispheres (Supplemental Table 1). Associations with DYX3 markers were conditioned on cortical thickness and volume measures of ROIs derived using the FreeSurfer software suite (http://surfer.nmr.mgh.harvard.edu/) (Supplemental Table 2) (Fischl 2012). DYX3 ROIs were selected to replicate previous associations in hippocampal and temporal cortical regions (Supplemental Table 2).

Associations of genotypes of interest with neuroimaging traits were tested by multiple regression analyses in R using the PING data portal (https://mmil-dataportal.ucsd.edu). To correct for the multiple ROIs, we set a statistical threshold of 0.05 divided by the number of ROIs tested for each imaging modality. For instance, a threshold of 0.003333 (0.05 / 15 regions of interest) was used for DYX2 associations of cortical thickness. A threshold of 0.003125 (0.05 / 16 regions of interest) was used for associations of DYX2 with FA and DYX3 with cortical thickness and volume. Associations were considered suggestive with *p* < 0.01. LD of genetic markers was calculated as D^’^ for all possible pairs of SNPs with Haploview v4.2 (Barrett et al. 2005).

## Results

The results of the genetic associations of DYX2 and DYX3 markers with imaging phenotypes are presented in Table 2 and in Supplemental Tables 3–6. The results from the DYX2 locus are presented first, followed by the DYX3 locus.

**Table 2:** Summary of associations (*p* < 0.01) of DYX2 and DYX3 markers with imaging measures

ROI	DYX2 markers	Gene/Locus	Measure	Effect	*p*-value^a^
Left pars opercularis	rs3777663	*ACOT13*	Thickness	0.03732	4.64 × 10^−3^
Left orbitofrontal	rs9461045	*KIAA0139*	Thickness	−0.0476	4.89 × 10^-4b^
Corpus callosum	rs9461045	*KIAA0139*	FA	−0.00842	5.89 × 10^−3^
Right All	rs9348646	*FAM65B*	FA	−0.00385	9.20 × 10^−3^
Left SLF	rs9348646	*FAM65B*	FA	−0.00576	4.61 × 10^−3^
Right SLF	rs9348646	*FAM65B*	FA	−0.00607	7.26 × 10^−3^
Left tSLF	rs9348646	*FAM65B*	FA	−0.00651	2.10 × 10^-3b^
Left pSLF	rs9348646	*FAM65B*	FA	−0.00527	1.00 × 10^−2^
ROI	DYX3 markers	Gene/Locus	Measure	Effect	*p*-value^a^
Left middle temporal	rs917235	DYX3^c^	Thickness	0.05897	3.96 × 10^−3^
Right Middle Temporal	rs2298948	*GCFC2*	Thickness	0.03175	5.65 × 10^−3^
Right Inferior Temporal	rs2298948	*GCFC2*	Volume	−548.75	7.21 × 10^−3^
Right Fusiform	rs6732511	DYX3^c^	Volume	478.22	3.15 × 10^−3^

*All* All Fiber Tracts, *SLF* Superior Longitudinal Fasiculus, *tSLF* Temporal Superior Longitudinal Fasiculus, *pSLF* Parietal Superior Longitudinal Fasiculus

^a^To correct for multiple testing with DYX2 markers, we used a threshold of 0.00333 for cortical thickness (0.05 divided by 15 regions of interest) and 0.003125 for FA (0.05 divided by 16 regions of interest). To correct for multiple testing with DYX3 markers, we used a threshold of 0.003125 for cortical thickness (0.05 divided by 16 regions of interest) and 0.002778 for cortical volume (0.05 divided by 18 regions of interest). Associations deemed suggestive if *p* < 0.01

^b^Survives correction for multiple testing as described above

^c^rs917235 and rs6732511 are located in the DYX3 locus upstream of *GCFC2* and *MRPL19*

### The DYX2 locus

Of the 8 DYX2 markers analyzed, 3 showed associations with neuroimaging phenotypes: rs9461045 in *KIAA0319*, rs3777663 in *ACOT13*, and rs9348646 in *FAM65B*. In this sample, the LD structure of the DYX2 locus suggests that rs9461045, rs3777663, and rs9348646 may be tagging the same genomic variation (Supplemental Figure 1). The strongest associations were seen with rs9461045 and cortical thickness in the left orbitofrontal region (*p* = 4.89 × 10^−4^) (Table 2). This association between the putative functional marker in *KIAA0319*, rs9461045, and left orbitofrontal cortical thickness persisted (*p* = 5.00 × 10^−3^) when average overall cortical thickness was included as a covariate in the model, suggesting specific effects of the marker in this region. There was suggestive association of rs3777663 in *ACOT13* with cortical thickness in the left pars opercularis (*p* = 4.64 × 10^−3^) but with no other ROI examined (*p* < 0.05) (Table 2, Supplemental Table 3). This association persisted when overall average cortical thickness was included as a covariate in the model (*p* = 4.0 × 10^−4^), indicating specific effects of *ACOT13* in the left pars opercularis region.

DYX2 associations with FA were suggestive and typically global in nature (Table 2, Supplemental Table 4). The strongest associations were seen with rs9461045 in *KIAA0319* and rs9348646 in *FAM65B*. These included associations of rs9461045 with FA in the corpus callosum (CC, *p* = 5.89 × 10^−3^) as well as rs9348646 with FA in the left superior longitudinal fasciculus (SLF) (*p* = 4.61 × 10^−3^) and right temporal SLF (tSLF) (*p* = 7.26 × 10^−3^) (Table 2). However, when overall FA was included as a covariate in these models, the associations with FA in these fiber tracts of interest were attenuated (*p* > 0.05), indicating that the suggestive effects of *KIAA0319* and *FAM65B* genotypes reflect global FA effects as opposed to specific regional effects (p > 0.05).

### The DYX3 locus

We examined the association of three DYX3 markers previously associated with various neurocognitive and imaging traits (Table 1). There were suggestive associations with neuroimaging phenotypes in temporal regions with all three markers. rs6732511 was in LD with both rs917235 and rs2298948; however, rs917235 and rs2298948 were not in LD with each other (Supplemental Figure 2). rs917235 and rs6732511 showed suggestive association with cortical thickness in the left middle temporal region (*p* = 3.96 × 10^−3^) and cortical volume in the right fusiform region (*p* = 3.15 × 10^−3^), respectively (Table 2, Supplemental Tables 5–6). There were also suggestive associations of rs2298248 with cortical thickness and volume in various temporal regions, including cortical thickness in the right middle temporal region (*p* = 5.65 × 10^−3^) and cortical volume in the right inferior temporal region (*p* = 7.21 × 10^−3^) (Table 2, Supplemental Tables 5–6). Notably, there were no associations of rs2298248 with hippocampal measures, as previously reported in the literature (Melville et al. 2012) (Supplemental Table 6).

## Discussion

The overall goal of this study was to gain biological insight into genetic markers that previously showed replicated associations with reading-, language-, and/or IQ-related traits. To do so, we interrogated markers within the DYX2 and DYX3 loci previously associated with neurobehavioral and neuroimaging traits. Within the DYX2 locus, there were associations of *KIAA0319* with cortical thickness in the left orbitofrontal region and *FAM65B* with global FA, with suggestive associations of *KIAA0319* with global FA and *ACOT13* with cortical thickness in the left pars opercularis region. These results suggest where and how DYX2 risk variants may give rise to their biological effects upon neurocognitive and language development. Additionally, we observed suggestive associations of DYX3 markers with cortical thickness and volume measures in temporal regions, further proposing a possible role of DYX3 risk elements (hypothesized to be *MRPL19* and/or *GCFC2*) in temporal regions. Notably, we did not replicate the associations between DYX3 markers and hippocampal volume, failing to confirm the findings of a previous study (Melville et al. 2012).

### Associations of DYX2 Genes *KIAA0319*, *ACOT13*, and *FAM65B*

Associations of DYX2 markers with cortical thickness and FA were limited to two genomic regions: *KIAA0319*/*ACOT13* and *FAM65B*. In our present and previous analyses, markers in the 5′ region of *KIAA0319*, including rs9461045, and rs3777663 in *ACOT13* are in high LD with each other and thus, appear to tag the same genomic locus (Eicher et al. 2014). The literature shows the minor allele of rs9461045 to be the risk allele, while our recent report suggests the minor allele of rs3777663 is protective (Dennis et al. 2009; Eicher et al. 2014). This was possibly mirrored by our imaging-genetic results. *KIAA0319* and *ACOT13* markers were associated with cortical thickness in the left orbitofrontal and left pars opercularis regions, respectively. The risk minor allele of rs9461045 in *KIAA0319* was associated with decreased cortical thickness, and the protective minor allele of rs3777663 in *ACOT13* associated with increased cortical thickness. Although the association signals of *KIAA0319* and *ACOT13* cannot be disentangled in this and other studies, the functional role of *KIAA0319* in neuronal migration makes it the likely effector gene (Newbury et al. 2011; Peschansky et al. 2010; Szalkowski et al. 2012, 2013; Centanni et al. 2014). These results, in conjunction with the literature, suggest that risk alleles of *KIAA0319* impair neuronal migration, resulting in reduced cortical thickness and then manifesting itself in poorer language and neurocognitive outcomes. Protective alleles, tagged by rs3777663, produce the opposite effects with ultimately improved language and neurocognitive skills. Future experimentation is needed to further demonstrate a direct link between genetic variant, neuronal migration, neuroimaging manifestation (in this case, cortical thickness), and the ultimate neurocognitive traits. Additionally, functional molecular work should discern whether *ACOT13* plays a role in neural phenotypes, particularly in the left orbitofrontal and pars opercularis regions.

Both *KIAA0319* and *FAM65B* were associated with overall FA, suggesting that risk alleles negatively impact the white matter integrity of fiber tracts. The global effects of these genes on FA suggest that *KIAA0319* and *FAM65B* have substantial impact on how brain circuits integrate various stimuli. In this PING sample, rs9461045 in *KIAA0319* and rs9348646 in *FAM65B* were in moderately strong LD with each other (D’ = 0.81), indicating that these SNPs may be tagging the same genomic variation (Supplemental Figure 1). However, our previous study of a larger sample of European children showed no evidence of LD between *KIAA0319* and *FAM65B* (Eicher et al. 2014). Future studies, particularly of the far less studied *FAM65B*, are needed to see how risk variants may influence myelination and/or neurophysiological properties throughout the entire brain.

### Suggestive associations of DYX3 markers

In this study, we show suggestive evidence of association for DYX3 markers with neuroimaging measures in temporal regions. Specifically, there were suggestive relationships between DYX3 markers in the middle and inferior temporal regions, as well as the fusiform gyrus. Previous work showed expression of the two hypothesized DYX3 risk genes, *MRPL19* and *GCFC2*, in these temporal regions (Anthoni et al. 2007). Temporal lobe functions have been well described and include auditory and visual processing, language comprehension, meaning derivation, and formation of new memories. Specifically, functional brain studies on individuals with RD have highlighted decreased activity in the left temporo-parietal region during both phonological processing tasks (Shaywitz et al. 1998; Temple et al. 2001) and simple speech tasks, with a notable increased level of activity in corresponding areas in the right brain in children with impaired reading and language skills (Breier et al. 2003; Brown et al. 2014). Additionally, previous work has shown decreased gray matter volume bilaterally in fusiform gyri in adolescents with RD (Kronbichler et al. 2008). In our present analysis, rs6732511 and rs2298948 were in LD and demonstrated suggestive association with cortical volume in the right fusiform gyrus. The literature shows the minor allele of rs6732511 to be protective (Anthoni et al. 2007), while the minor allele of rs2298948 has been shown to be the risk allele (Melville et al. 2012). Here, the risk minor allele of rs2298948 was associated with decreased cortical volume possibly yielding adverse language and neurocognitive outcomes, while the protective minor allele of rs6732511 associated with increased cortical volume possibly giving rise to positive language and neurocognitive outcomes. Future studies, using other independent methods, such as animal models and/or longitudinal human neuroimaging strategies, are necessary to confirm a direct relationship between risk marker, neuroimaging observation, and neurocognitive outcome.

Notably, we did not observe associations of rs2298948 with cortical volume measures in the hippocampus, as was observed in a GWAS of hippocampal volume (Melville et al. 2012). The lack of replication of this GWAS signal does not necessarily mean that *GCFC2* does not contribute to hippocampal volume, as the present study substantially differs in terms of age (pediatric versus adult populations). Little is known of the possible neural function of the DYX3 candidate genes *MRPL19* and *GCFC2*. In order to further support each of their roles in RD, LI, and neurocognition, functional and animal-based work should be completed specifically interrogating their cellular and neural contributions.

### Use of imaging-genetics in neurocognitive traits

Currently, we have an incomplete view of the biological etiologies underlying RD, LI, and related neurocognitive traits. Neuropsychological, genetic, molecular, and imaging studies have made much progress into identifying the specific impairments, candidate genes/signatures, and possible pathways that may contribute to the deficits observed in dyslexia and related disorders. However, how these varying levels of phenotype interact and relate to each other to lead to the ultimate neurocognitive phenotype remains elusive. Imaging-genetics studies can suggest a more mechanistic understanding into the pathophysiology of these disorders, and provide an ethical means to gain mechanistic insight into the pathophysiology in human subjects in vivo. Instead of solely relying upon animal models that may approximate the behavioral and biological deficits seen in RD and LI, human imaging-genetics allow for the direct examination of human risk genetic variants with imaging data directly related to reading and language processes. Additionally, neuroimaging traits may represent measureable phenotypes closer to the underlying biology seen in behavior and cognition. Therefore, finding genetic associations with phenotypes closer to the underlying biology, in this case neuroimaging, may be more readily detectable than those conditioned on neurocognitive and language measures (Thompson et al. 2010). The neuroimaging consequences of risk variants can also inform mechanistic studies in regards to where and how neurological dysfunction occurs. However, interactive effects with other genetic and environmental factors must be taken into account to effectively uncover the underlying mechanisms of these traits.

### Limitations

There are limitations in the presented imaging-genetic analyses. First, discerning whether these neuroimaging phenotypes are causal of or resulting from language capabilities is challenging due to the inherent complexity and plasticity of the brain. Further neurophysiological and molecular interrogation using cell-based and organismal models, as well as longitudinal imaging-genetic studies, can help in determining causal and temporal relationships. Second, interpreting what imaging data and their associations with genetic and behavioral factors actually mean in a biological context is challenging. Although there are numerous hypotheses regarding the actual meaning of FA and structural measures through DTI and sMRI, it is still difficult to make definitive conclusions about the biological and behavioral implications of these data. Third, our analyses started with a small subset of genetic variants and brain ROIs. We then examined the implications of these specific genetic markers on brain imaging measures in specific brain regions. Selection bias, along with incomplete coverage of associated genes and the rest of the genome, could lead to misleading and incomplete results and hypotheses. Future studies could use neuroimaging phenotypes as an endophenotype to condition GWAS, sequencing, and voxel-based analyses in order to discover novel genes and neuroimaging traits that contribute to reading, language, and cognition. Additionally, our cross-sectional, unselected sample permitted us only to examine general, quantitative performance as opposed to case-control differences. The lack of case-control analyses may explain the absence of *DCDC2* associations, as *DCDC2* tends to generally be associated with severe case-control phenotypes (Meng et al. 2005; Powers et al. 2013). Future samples with different recruitment strategies and/or large sample size should examine imaging-genetic associations between these ROIs and markers of interest. Lastly, we limited our sample size to those of European ancestry as this study was a direct follow-up of genetic studies in European samples (Eicher et al. 2014; Scerri et al. 2012). This limited sample size and the allele frequencies of the examined SNPs (Table 1) prevented us for making meaningful inferences on age and gene-by-age effects on brain development as shown in other PING studies (Brown et al. 2012; Douet et al. 2014). Identification of functional SNPs and variants in the DYX2 and DYX3 regions as well as studies of these SNPs in non-European samples will enable us to examine these gene-by-age effects with greater confidence and accuracy.

## Conclusion

In conclusion, this study aimed to interrogate the neuroimaging consequences of genetic markers that had shown replicated associations with reading-, language-, and/or IQ-related traits. In our analyses of the DYX2 locus, we observed associations of *KIAA0319*, *ACOT13*, and *FAM65B* with cortical thickness and/or FA. We also observed suggestive associations of DYX3 markers with cortical thickness and volume measures within temporal regions. These associations offer insight into how these risk genetic markers may give rise to deficits in reading, verbal language, and IQ. Future studies should further interrogate these neurological phenotypes by using cellular, organismal, and molecular models. These studies could further connect risk genetic variants, to cellular phenotypes, to neuroimaging alterations, and to the ultimate deficits in language and communication.

## Electronic supplementary material

Below is the link to the electronic supplementary material.

Supplemental Table 1 (DOCX 33 kb)

Supplemental Table 2 (DOCX 31 kb)

Supplemental Table 3 (DOCX 38 kb)

Supplemental Table 4 (DOCX 40 kb)

Supplemental Table 5 (DOCX 48 kb)

Supplemental Table 6 (DOCX 51 kb)

Supplemental Figure 1 (DOCX 32 kb)

Supplemental Figure 2 (DOCX 26 kb)

## Abbreviations

RD: Reading disability
LI: Language impairment
SSD: Speech sound disorder
LD: Linkage disequilibrium
GWAS: Genome-wide association study
fMRI: Functional magnetic resonance imaging
sMRI: Structural magnetic resonance imaging
FA: Fractional anisotropy
DTI: Diffusion tensor imaging
PING: Pediatric imaging neurocognition genetics
UCSD: University of California at San Diego
ROI: Region of interest
GAF: Genetic ancestry factor
CC: Corpus callosum
SLF: Superior longitudinal fasciculus
tSLF: Temporal superior longitudinal fasciculus
