# Supplementary material for: Dyslexia and language impairment associated genetic markers influence cortical thickness and white matter in typically developing children
Source: Brain Imaging Behav. 2015 May 9;10:272–82. doi: 10.1007/s11682-015-9392-6 (PMC4639472; doi:10.1007/s11682-015-9392-6)
Supplement: Supplementary file 1 — (DOCX 33 kb) [file 11682_2015_9392_MOESM1_ESM.docx]

Supplemental Table 1: Fiber tracts and cortical regions of interest examined in DYX2 analyses within the PING cohort for fractional anisotropy (FA) and cortical thickness, respectively

| Fiber Tract | | Cortex | |
| --- | --- | --- | --- |
| Region of Interest (ROI) | Hemisphere | Region of Interest (ROI) | Hemisphere |
| All Fiber Tracts | Bilateral, Right, and Left | Occipital | Right and Left |
| Inferior Longitudinal Fasciculus (ILF) | Right and Left | Anteromedial Temporal | Right and Left |
| Inferior Fronto-occipital Fasciculus (IFO) | Right and Left | Posterolateral Temporal | Right and Left |
| Superior Longitudinal Fasciculus (SLF) | Right and Left | Superior Parietal | Left |
| Temporal Superior Longitudinal Fasciculus (tSLF) | Right and Left | Orbitofrontal | Left |
| Parietal Superior Longitudinal Fasciculus (pSLF) | Right and Left | Superior Temporal | Left |
| Striatal Inferior Frontal Cortex (SIFC) | Right and Left | Inferior Parietal | Left |
| Corpus Callosum (CC) | Bilateral | Dorsomedial Frontal | Left |
|  |  | Precuneus | Left |
|  |  | Dorsolateral Prefrontal | Left |
|  |  | Pars Opercularis | Left |
|  |  | Central | Left |
