# Supplementary material for: Dyslexia and language impairment associated genetic markers influence cortical thickness and white matter in typically developing children
Source: Brain Imaging Behav. 2015 May 9;10:272–82. doi: 10.1007/s11682-015-9392-6 (PMC4639472; doi:10.1007/s11682-015-9392-6)
Supplement: Supplementary file 2 — (DOCX 31 kb) [file 11682_2015_9392_MOESM2_ESM.docx]

Supplemental Table 2: Cortical regions of interest examined in DYX3 analyses in the PING cohort

| **Region of Interest** | **Hemisphere** | **Thickness** | **Volume** | **DYX3 markers** |
| --- | --- | --- | --- | --- |
| Inferior Temporal | Right/Left | Yes | Yes | All |
| Middle Temporal | Right/Left | Yes | Yes | All |
| Superior Temporal | Right/Left | Yes | Yes | All |
| Temporal Pole | Right/Left | Yes | Yes | All |
| Transverse Temporal | Right/Left | Yes | Yes | All |
| Fusiform | Right/Left | Yes | Yes | All |
| Parahippocampal | Right/Left | Yes | Yes | All |
| Lingual | Right/Left | Yes | Yes | All |
| Hippocampus | Right/Left | No | Yes | Only rs2298948 |
