# Supplementary material for: Dyslexia and language impairment associated genetic markers influence cortical thickness and white matter in typically developing children
Source: Brain Imaging Behav. 2015 May 9;10:272–82. doi: 10.1007/s11682-015-9392-6 (PMC4639472; doi:10.1007/s11682-015-9392-6)
Supplement: Supplementary file 3 — (DOCX 38 kb) [file 11682_2015_9392_MOESM3_ESM.docx]

Supplemental Table 3: Associations of rs9461045 in *KIAA0139* and rs3777663 in *ACOT13* with average cortical thickness

|  | **rs9461045** | | **rs3777663** | |
| --- | --- | --- | --- | --- |
| **Region of Interest** | **Slope** | **p-value** | **Slope** | **p-value** |
| Right Occipital | -0.0127 | 0.334 | -0.00453 | 0.719 |
| Left Occipital | -0.014 | 0.274 | 0.00364 | 0.766 |
| Right Anteromedial Temporal | -0.0198 | 0.166 | 0.00003 | 0.998 |
| Left Anteromedial Temporal | -0.0219 | 0.122 | 0.00513 | 0.707 |
| Right Posterolateral Temporal | -0.0005 | 0.973 | 0.00257 | 0.853 |
| Left Posterolateral Temporal | -0.0057 | 0.687 | 0.02056 | 0.1301 |
| Left Superior Parietal | -0.0154 | 0.314 | 0.01892 | 0.196 |
| Left Orbitofrontal | -0.0476 | 0.000489** | -0.00936 | 0.456 |
| Left Superior Temporal | -0.0271 | 0.072 | 0.01163 | 0.421 |
| Left Inferior Parietal | -0.0271 | 0.0764 | 0.0111 | 0.448 |
| Left Dorsomedial Frontal | -0.0233 | 0.15 | 0.00457 | 0.769 |
| Left Precuneus | -0.0237 | 0.0769 | 0.00422 | 0.742 |
| Left Dorsolateral Prefrontal | -0.0262 | 0.098 | 0.015 | 0.325 |
| Left Pars Opercularis | -0.0291 | 0.0348* | 0.03732 | 0.00464** |
| Left Central | -0.0091 | 0.562 | 0.01642 | 0.277 |

*p<0.05 **p<0.01
