# Supplementary material for: Dyslexia and language impairment associated genetic markers influence cortical thickness and white matter in typically developing children
Source: Brain Imaging Behav. 2015 May 9;10:272–82. doi: 10.1007/s11682-015-9392-6 (PMC4639472; doi:10.1007/s11682-015-9392-6)
Supplement: Supplementary file 4 — (DOCX 40 kb) [file 11682_2015_9392_MOESM4_ESM.docx]

Supplemental Table 4: Associations of rs9461045 in *KIAA0139* and rs9348646 in *FAM65B* with fractional anisotropy (FA) measures

|  | **rs9461045** | | **rs9348646** | |
| --- | --- | --- | --- | --- |
| **Region of Interest** | **Slope** | **p-value** | **Slope** | **p-value** |
| All | -0.00423 | 0.0316* | -0.00346 | 0.01767* |
| Right All | -0.00448 | 0.0247* | -0.00385 | 0.0092** |
| Left All | -0.00413 | 0.0427* | -0.00311 | 0.0397* |
| Right ILF | -0.0013 | 0.656 | -0.00429 | 0.0466* |
| Left ILF | -0.00204 | 0.504 | -0.00376 | 0.096 |
| Right IFO | -0.00098 | 0.723 | -0.00481 | 0.0182* |
| Left IFO | -0.00109 | 0.715 | -0.00161 | 0.466 |
| Right SLF | -0.00566 | 0.0508 | -0.00413 | 0.0548 |
| Left SLF | -0.00601 | 0.0286* | -0.00576 | 0.00461** |
| Right tSLF | -0.00489 | 0.11 | -0.00607 | 0.00726** |
| Left tSLF | -0.00665 | 0.02* | -0.00651 | 0.0021** |
| Right pSLF | -0.00597 | 0.0387* | -0.00369 | 0.0852 |
| Left pSLF | -0.00481 | 0.0828 | -0.00527 | 0.01* |
| Right SIFC | -0.00199 | 0.455 | -0.00301 | 0.127 |
| Left SIFC | 0.00079 | 0.771 | -0.00046 | 0.818 |
| CC | -0.00842 | 0.00589** | -0.00547 | 0.0161* |

*p<0.05 **p<0.01

Abbreviations: All (All Fiber Tracts), ILF (Inferior Longitudinal Fasiculus), IFO (Inferior Fronto-occipital Fasiculus), SLF (Superior Longitudinal Fasiculus), tSLF (Temporal Superior Longitudinal Fasiculus), pSLF (Parietal Superior Longitudinal Fasiculus), SIFC (Striatal Inferior Frontal Cortex), CC (Corpus Callosum)
