# Supplementary material for: Dyslexia and language impairment associated genetic markers influence cortical thickness and white matter in typically developing children
Source: Brain Imaging Behav. 2015 May 9;10:272–82. doi: 10.1007/s11682-015-9392-6 (PMC4639472; doi:10.1007/s11682-015-9392-6)
Supplement: Supplementary file 5 — (DOCX 48 kb) [file 11682_2015_9392_MOESM5_ESM.docx]

Supplemental Table 5: Association of rs917235 in DYX3 with cortical thickness in temporal regions

|  | **rs917235** | | **rs2298948** | |  |
| --- | --- | --- | --- | --- | --- |
| **Region of Interest** | **Slope** | **p-value** | **Slope** | **p-value** | |
| Right Inferior Temporal | -0.00289 | 0.802 | 0.0078 | 0.6451 | |
| Left Inferior Temporal | -0.0148 | 0.184 | -0.00266 | 0.8701 | |
| Right Middle Temporal | 0.02775 | 0.0146* | 0.0317 | 0.00565** | |
| Left Middle Temporal | 0.02383 | 0.00396** | -0.0152 | 0.4148 | |
| Right Superior Temporal | 0.0105 | 0.32313 | -0.0015 | 0.9245 | |
| Left Superior Temporal | 0.0172 | 0.1367 | 0.00441 | 0.7939 | |
| Right Temporal Pole | -0.0138 | 0.5028 | -0.03375 | 0.2617 | |
| Left Temporal Pole | 0.00204 | 0.925 | -0.0535 | 0.0885 | |
| Right Transverse Temporal | 0.03297 | 0.0489* | 0.0386 | 0.1191 | |
| Left Transverse Temporal | -0.00803 | 0.658 | 0.0109 | 0.6788 | |
| Right Fusiform | 0.000255 | 0.998 | 0.00608 | 0.6837 | |
| Left Fusiform | 0.000616 | 0.9509 | -0.00513 | 0.7267 | |
| Right Parahippocampal | -0.0168 | 0.3531 | 0.024 | 0.3616 | |
| Left Parahippocampal | -0.00722 | 0.7101 | -0.0087 | 0.7585 | |
| Right Lingual | 0.00489 | 0.628 | 0.0294 | 0.0464 | |
| Left Lingual | 0.00172 | 0.864 | 0.00914 | 0.537 | |

*p<0.05 **p<0.01
