# Supplementary material for: Dyslexia and language impairment associated genetic markers influence cortical thickness and white matter in typically developing children
Source: Brain Imaging Behav. 2015 May 9;10:272–82. doi: 10.1007/s11682-015-9392-6 (PMC4639472; doi:10.1007/s11682-015-9392-6)
Supplement: Supplementary file 6 — (DOCX 51 kb) [file 11682_2015_9392_MOESM6_ESM.docx]

Supplemental Table 6: Association of rs2298948 and rs6732511 with cortical volume in the temporal lobe and hippocampus

|  | **rs2298948** | | **rs6732511** | |
| --- | --- | --- | --- | --- |
| **Region of Interest** | **Slope** | **p-value** | **Slope** | **p-value** |
| Right Inferior Temporal | -548.75 | 0.00721** | 112.109 | 0.6292 |
| Left Inferior Temporal | -379.33 | 0.084 | 344.21 | 0.166 |
| Right Middle Temporal | 141.21 | 0.494 | 513.960 | 0.0275* |
| Left Middle Temporal | --152.4 | 0.462 | 341.3 | 0.1578 |
| Right Superior Temporal | -40.11 | 0.805 | 233.501 | 0.2033 |
| Left Superior Temporal | -24.96 | 0.899 | 39.05 | 0.860 |
| Right Temporal Pole | 7.011 | 0.861 | -100.363 | 0.0250* |
| Left Temporal Pole | 6.961 | 0.872 | -99.27 | 0.0427* |
| Right Transverse Temporal | -2.469 | 0.921 | 28.725 | 0.3079 |
| Left Transverse Temporal | -9.735 | 0.754 | 16.406 | 0.6396 |
| Right Fusiform | -271.73 | 0.096 | 525.41 | 0.00315** |
| Left Fusiform | 79.41 | 0.654 | -70.36 | 0.7254 |
| Right Parahippocampal | 21.41 | 0.592 | -29.134 | 0.5189 |
| Left Parahippocampal | -13.493 | 0.698 | 19.820 | 0.6148 |
| Right Lingual | 45.73 | 0.719 | 70.41 | 0.6232 |
| Left Lingual | --132.6 | 0.318 | 194.052 | 0.1963 |
| Right Hippocampus^a^ | -9.322 | 0.819 |  |  |
| Left Hippocampus^a^ | -0.275 | 0.995 |  |  |

^a^subcortical volume *p<0.05 **p<0.01
