# Supplementary material for: Dyslexia and language impairment associated genetic markers influence cortical thickness and white matter in typically developing children
Source: Brain Imaging Behav. 2015 May 9;10:272–82. doi: 10.1007/s11682-015-9392-6 (PMC4639472; doi:10.1007/s11682-015-9392-6)
Supplement: Supplementary file 7 — (DOCX 32 kb) [file 11682_2015_9392_MOESM7_ESM.docx]

**Supplemental Figures**


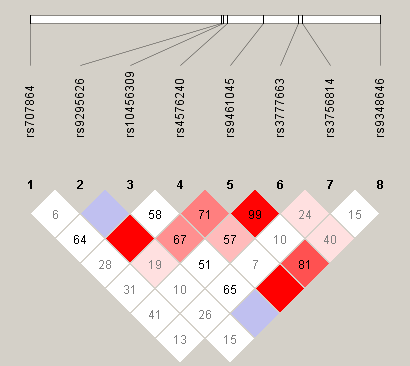


Supplemental Figure 1: Linkage disequilibrium (LD) structure of the 8 DYX2 markers examined in the PING cohort as measured by D’.
